# Systematic review of comparing single-incision versus conventional laparoscopic right hemicolectomy for right colon cancer

**DOI:** 10.1186/s12957-019-1721-6

**Published:** 2019-11-04

**Authors:** Xin Liu, Wei-hong Yang, Zhou-guang Jiao, Ji-fu Zhang, Rui Zhang

**Affiliations:** 1 Department of Colorectal Surgery, Cancer Hospital of China Medical University, Liaoning Cancer Hospital and Institute, No 44 Xiaoheyan Road, Dadong District, Shenyang, 110042 Liaoning Province People’s Republic of China; 2 Institute of Physical Science and Information Technology, Anhui University, Hefei, 230601 Anhui Province People’s Republic of China; 3 State Key Laboratory of Pathogen and Biosecurity, Beijing Institute of Microbiology and Epidemiology, Beijing, 100071 People’s Republic of China

**Keywords:** Single incision, Conventional laparoscopic right hemicolectomy, Right colon cancer, Meta-analysis

## Abstract

**Background:**

Single-incision laparoscopic right hemicolectomy (SILS) has long used in surgery for a long time. However, there is barely a systemic review related to the comparison between the SILS and the conventional laparoscopic right hemicolectomy (CLS) for the right colon cancer in the long term follow-up. Herein, we used the most recent articles to compare these two techniques by meta-analysis.

**Methods:**

We searched PubMed, Web of Science, Cochrane Library and Wanfang databases to compare SILS with CLS for right colon cancer up to May 2019. The operative, postoperative, pathological and mid-term follow-up outcomes of nine studies were extracted and compared.

**Results:**

A total of 1356 patients participated in 9 studies, while 653 patients were assigned to the SILS group and 703 patients were assigned to the CLS group. The patients’ baselines in the SILS group were consistent with those in the CLS group. Compared to the CLS group, the SILS group had a shorter operation duration (SMD − 23.49, 95%CI − 36.71 to − 10.27, *P* < 0.001, chi-square = 24.11), shorter hospital stay (SMD − 0.76, 95% `CI − 1.07 to − 0.45, *P* < 0.001, chi-square = 9.85), less blood loss (SMD − 8.46, 95% CI − 14.59 to − 2.34; *P* < 0.05; chi-square = 2.26), smaller incision length (SMD − 1.60, 95% CI − 2.66 to − 0.55, *P* < 0.001; chi-square = 280.44), more lymph node harvested (SMD − 0.98, 95% CI − 1.79 to − 0.16, *P* < 0.05; chi-square = 4.61), and a longer proximal surgical edge (SMD − 0.51, 95% CI − 0.93 to − 0.09, *P* < 0.05; chi-square = 2.42). No significant difference was found in other indexes. After we removed a single large study, we performed another meta-analysis again. The operation duration in the SILS group was still better than that in the CLS group.

**Conclusion:**

SILS could be a faster and more reliable approach than CLS for the right colon cancer and could accelerate patient recovery, especially for patients with a low BMI.

## Background

Conventional laparoscopic colectomy was reported by Jacobs in 1991. This procedure became increasingly popular in the clinic. Conventional laparoscopic right hemicolectomy (CLS) can decrease postoperative pain and accelerate patient recovery [1]. Additionally, laparoscopic trocar can cause severe trauma to the abdominal wall and hemorrhage at the port site. Single-incision laparoscopic right hemicolectomy (SILS) was invented by Remzi in 2008 [2]. A single incision can complete the operation and facilitate the removal of the specimen. According to the previous studies on SLIS and CLS, SILS has some advantages in safety and recovery [3–5]. However, there have been only two meta-analyses comparing SILS with CLS so far. These analyses were reported by Vettoretto et al. and Dong et al. in 2013 and 2018 respectively. Vettoretto et al. found no significant difference between the two techniques [6]. Dong et al. mainly focused on right colon diseases (including Crohn’s disease, polyp, and inflammation) and found that SILS had the advantages related to incision length, hospital stay and blood loss [7].

However, considering SILS and CLS, which surgical method is more suitable for the treatment of right colon cancer? Does SILS have more advantages than CLS in right colon cancer treatment? Could SILS replace CLS to treat right colon cancer? In this paper, we will focus on the right colon cancer and perform a meta-analysis to evaluate the effects of SILS and CLS on patients with right colon cancer.

## Methods

### Literature search

We performed this study according to the PRISMA guidelines (Preferred Reporting Items for Systematic Reviews and Meta-Analysis). The criteria for the guidelines included patients, intervention, comparator, outcomes and study design (PICOS). Population: patients with right colon cancer; Intervention: SILS or CLS; Comparator: clinical and pathological outcomes of two methods; Outcomes: clinical and long-term outcomes were expressed as the standardized mean differences (SMD) with 95% confidence intervals (95% CIs) for continuous data and relative risks (ORs or RRs) with 95% CIs for dichotomous outcomes.

We systematically searched all the useful studies in PubMed, Medline, Cochrane Library and Wanfang databases up to May 2019. The search terms are “single-incision” OR “single-site” OR “single-port” AND “colon cancer” OR “right colon cancer” AND “laparoscopic surgery”. The detail strategies from PubMed are shown in Additional file 1: Table S1.

According to our search strategy, we retained relevant literature and eliminated the duplicated records (Identification). After reading the titles and abstracts carefully, we removed the literature, which was inconsistent with our research (Screening). Based on the inclusion and exclusion criteria, we removed the non-NRCT and obtained the final studies for our research (Eligibility). We tried our best to obtain the most comprehensive information of the related articles. We removed duplicated studies, and studies with no SILS, CLS or laparoscopic approaches, and only focused on the right colon cancer. Reviews, letters, meetings, case reports, and no clinical controlled studies were excluded.

### Inclusion and exclusion criteria

The studies were reviewed carefully according to the criteria as follows. The inclusion criteria were as follows: (1) the outcomes between SILS and CLS for right hemicolectomy in right colon cancer were compared, (2) only studies written in English were collected, and (3) RCNTs (retrospective comparative non-randomized studies), PCNTs (prospective comparative non-randomized studies), and comparative observational (cohort and case-control) studies were collected.

The exclusion criteria were as follows: (1) studies from the same center should be removed to avoid data duplication, (2) reports without a distinct group of right hemicolectomy in right colon diseases, or (3) the main outcomes were not clearly reported.

### Data extraction and quality control

The literature was independently searched according to the rules of the Newcastle-Ottawa Scale (NOS) by two reviewers (XL and JXZ) independently [8]. The following data are shown in Table 1, including study characteristics (the first author, publication data, study area, study type, size, and study quality) and patient baselines (age, gender, BMI, previous abdominal surgery, clinical stage, and anastomosis type). The incision length in the CLS group was the sum of all incisions. A third reviewer resolved all disagreements about the articles until a consensus was reached. We contacted the authors of the included studies with incomplete data but did not receive any additional information.

**Table 1:** Characteristics of the included studies in the meta-analysis

Study	Year	Country	Study type	Patients (*n*)	Age	Sex (M/F)		NOS stars
				SILS/CLS	SILS/CLS	SILS/CLS		
Curro [9]	2012	Italy	R	10/10	60/59	4/6	3/7	6
Yun [10]	2013	Korea	R	66/93	61/59	33/33	58/35	7
Takemasa [11]	2014	Japan	P	69/69	65/66	31/38	36/33	7
Suzuki [12]	2016	Japan	R	35/35	68/69	21/14	14/21	7
Yu [13]	2016	Korea	P	38/92	63/65	21/19	45/47	8
Tokudka [14]	2016	Japan	R	27/36	77/77	16/11	15/21	6
Chouillard [15]	2016	France	R	336/256	61/65	160//176	128/128	8
Kim [16]	2017	Korea	R	40/80	66/63	22/28	50/30	7
Song [17]	2018	China	R	32/32	59/65	16/16	16/16	7
Study	BMI		Previous abdominal surgery (n)	Clinical stage			Anastomosis type	
	SILS/CLS		SILS/CLS	SILS (I/II/III/IV)	CLS (I/II/III/IV)			
Curro [9]	25/26		2/3	NR	NR		End to end	
Yun [10]	23.8/24.2		13/23	22/24/20/0	32/31/30/0		NR	
Takemasa [11]	21.5/22.2		16/19	32/23/14/0	31/21/17/0		End to end	
Suzuki [12]	26.2/25.0		14/12	7/12/16/0	8/10/17/0		NR	
Yu [13]	24.6/24.3		8/31	NR	NR		NR	
Tokudka [14]	23.1/21.9		7/9	NR	NR		End to end	
Chouillard [15]	27/26.2		56/56	NR	NR		End to side	
Kim [16]	23.5/23.4		9/19	17/14/9/0	34/30/16/0		End to side	
Song [17]	22.7/22.8		11/9	9/13/10/0	8/14/10/0		End to side	

*F* female, *M* male, *NR* No record, *R* retrospective nonrandomized controlled trials, *P* prospective comparative nonrandomized trial, *SILS* single-port laparoscopic right hemicolectomy, *CLS* conventional laparoscopic right hemicolectomy, *NOS stars* modified Newcastle Ottawa Scale

### Statistical analysis

We used Revman 5.0 and Stata 11.0 to perform a meta-analysis by the standardized mean differences (SMD) with 95% confidence intervals (95% CIs) for continuous data and relative risks (ORs or RRs) with 95% CIs for dichotomous outcomes. The statistical heterogeneity was estimated by *I*^*2*^ statistic and chi-square test. Begg’s test and funnel plots were used to evaluate publication bias. When *I*^*2*^ > 50% and Begg’s test and the funnel plots indicated publication bias, random effects models were used. When *I*^*2*^ < 50% and the funnel plot and Begg’s test showed no publication bias, a fixed effects model was used. *P* < 0.05 indicated significant differences. A sensitivity analysis was conducted to decrease the impact of a single study.

## Results

### Study selection

**Fig. 1:**
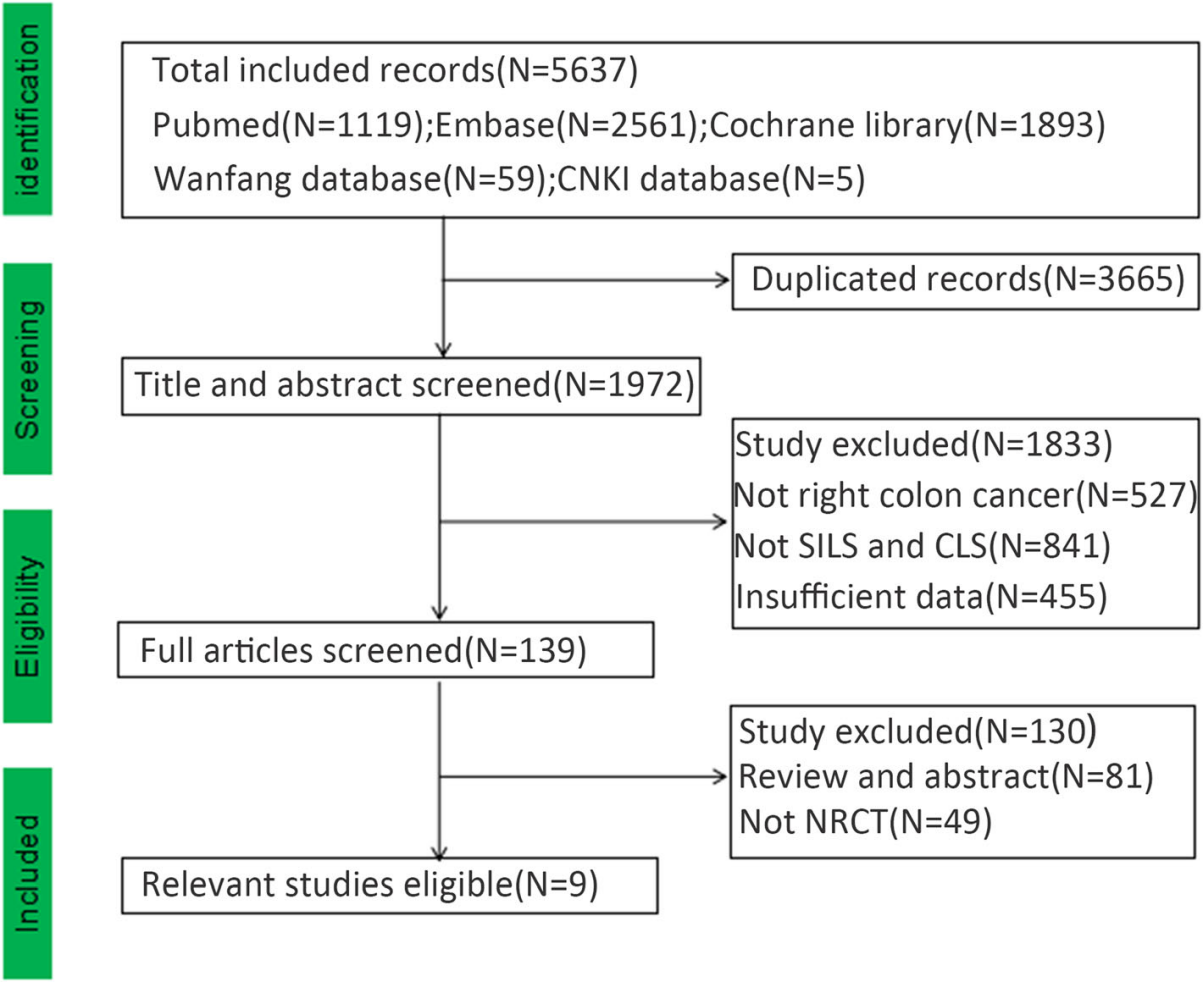
Flowchart of the included studies

We found 5637 related publications related to SILS and CLS from the database and deleted 3665 duplicated records. After we reviewed over all the titles and abstracts carefully, 1833 studies were deleted due to no SILS, CLS, or right colon cancer. Finally, a total of 1356 patients with right colon cancer in 9 English studies were included [9–17] (Fig. 1). The SILS group had 653 patients and the CLS group had 703 patients (Table 1). There was no significant difference in the baseline characteristics of patients (age, gender, BMI, ratio of pervious surgery, and clinical stage) of the patients.

### Quality assessment results

The quality was estimated according to the modified Newcastle Ottawa Scale (NOS). The study quality was low for 1 < scores ≤ 3, moderate for 4 < scores ≤ 6, and high for 7 ≤ scores ≤ 9. Seven studies had achieved scores of 7 to 8 with high quality, while other studies had moderate quality. The included NRCTs all had moderate- to high-grade quality, and the specific scores are shown in Table 1. This study only included two prospective clinical trials. We did not find any RCTs related to this study after searching many studies.

### Operative data

#### Operation duration, blood loss, and incision length

**Fig. 2:**
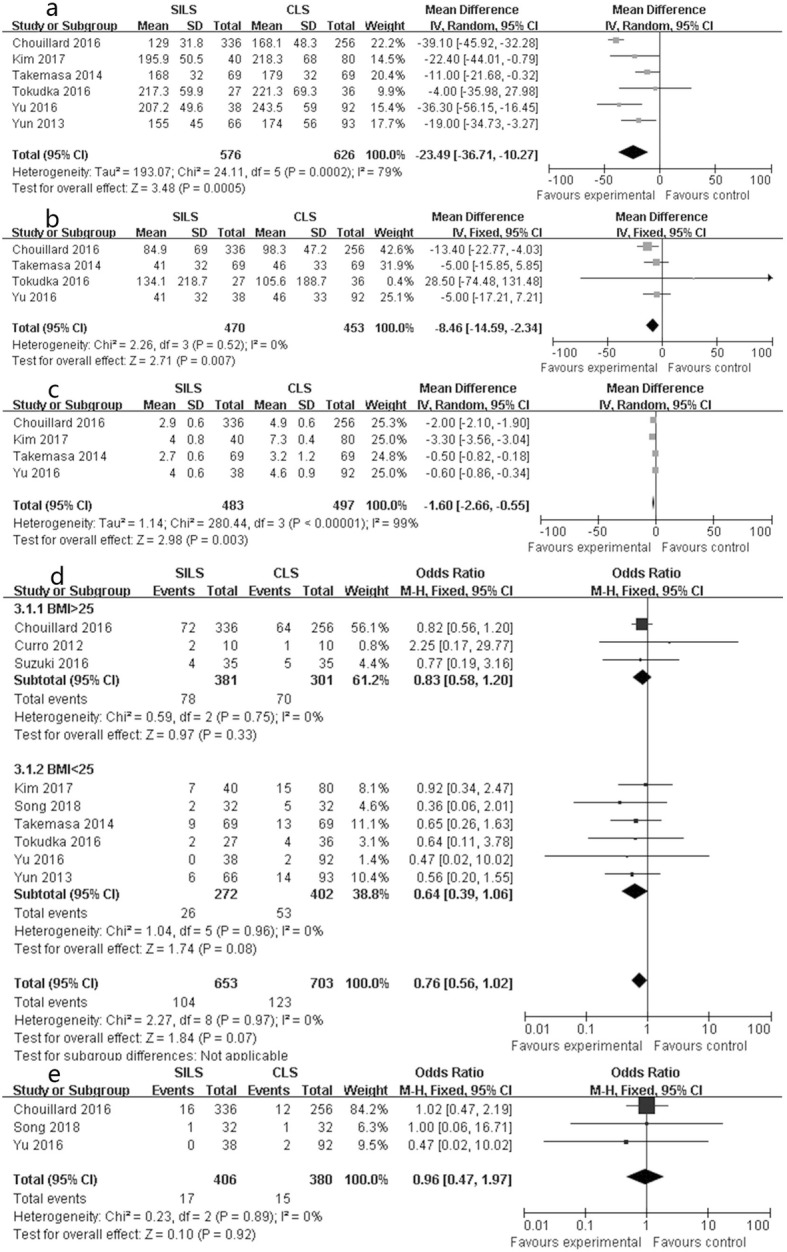
Forest plot of operative outcomes: **a** operation time, **b** blood loss, **c** incision length, **d** postoperative complication, **e** postoperative complication (C-D grade)

Six studies reported complete data on operation duration. SILS was associated with significantly less operation time than CLS in the random effects model (SMD − 23.49, 95% confidence interval [CI] − 36.71 to − 10.27, *P* < 0.001, chi-square = 24.11, *P* < 0.001, *I*^2^ = 79.8%, Fig. 2a).

Depending on the available data from four studies, SILS had less blood loss than CLS without significant heterogeneity in the fixed effects model (SMD − 8.46, 95% CI − 14.59 to − 2.34; *P* < 0.05; chi-square = 2.26, *P* = 0.5, *I*^2^ = 0%, Fig. 2b) (Table 2).

**Table 2:** Comparison of operative data between SILS and CLS for the included studies

Study	Operation time (min)		Blood loss (ml)		Incision length (cm)		Complication		Complication (C-D grade)	
	SILS	CLS	SILS	CLS	SILS	CLS	SILS	CLS	SILS	CLS
Curro [9]	170	160	35	50	4.3	4.5	2	1	NR	NR
Yun [10]	155 ± 45	174 ± 56	NR	NR	NR	NR	6	14	0	2
Takemasa [11]	168 ± 32	179 ± 32	41 ± 32	46 ± 33	2.7 ± 0.6	3.2 ± 1.2	9	13	NR	NR
Suzuki [12]	167	162	26	23	4	4.5	4	5	NR	NR
Yu [13]	207.2 ± 49.6	243.5 ± 59.0	128.5 ± 85.1	162.8 ± 56.5	4.0 ± 0.6	4.6 ± 0.9	0	2	NR	NR
Tokudka [14]	217.3 ± 59.9	221.3 ± 69.3	134.1 ± 218.7	105.6 ± 188.7	NR	NR	2	4	NR	NR
Chouillard [15]	129.0 ± 31.8	168.1 ± 48.3	84.9 ± 69.0	98.3 ± 47.2	2.9 ± 0.63	4.9 ± 0.6	72	64	16	12
Kim [16]	195.9 ± 50.5	218.3 ± 68.0	75	50	4.0 ± 0.8	7.3 ± 0.4	7	15	NR	NR
Song [17]	175	145	65	100	4	7	2	5	1	1

*SILS* single-port laparoscopic right hemicolectomy, *CLS* conventional laparoscopic right hemicolectomy, *NR* no record

SILS had a shorter incision length than CLS in the random effects model with high heterogeneity (SMD − 1.60, 95% CI − 2.66 to − 0.55, *P* < 0.001; chi-square = 280.44, *P* < 0.001, *I*^2^ = 99%; Fig. 2c).

#### Postoperative complication and postoperative complication (C-D grade)

All included studies reported postoperative complication and three studies reported postoperative complication (C-D grade). There was no significant difference in postoperative complication (OR 0.76, 95% CI 0.56 to 1.02, *P* = 0.07; chi-square = 2.27, *P* = 0.97, *I*^2^ = 0%, Fig. 2d) or postoperative complication (C-D grade) (OR 0.96, 95% CI 0.47 to 1.97, *P* = 0.92; chi-square = 0.23, *P* = 0.89, *I*^2^ = 0%, Fig. 2e) without statistical heterogeneity. The subgroup analysis revealed that postoperative complication was not associated with BMI.

### Postoperative data

#### Conversion rates and hospital stay

**Fig. 3:**
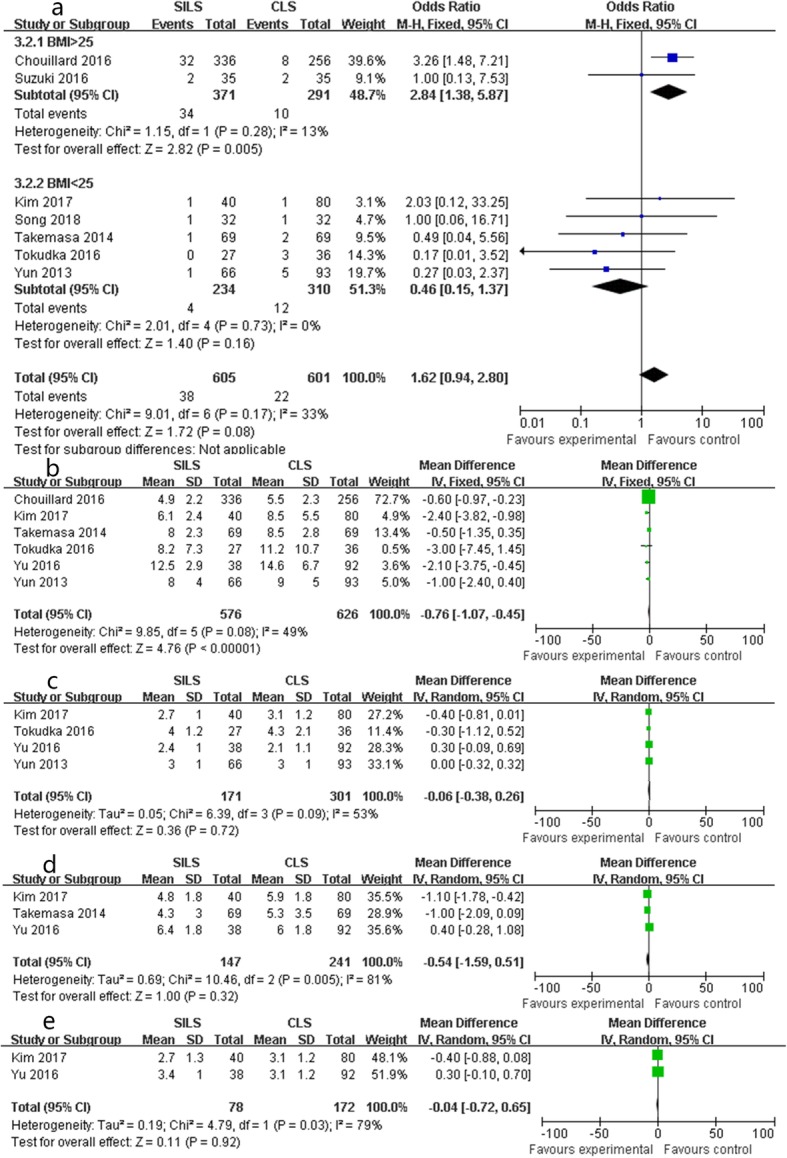
Forest plot of recovery outcomes: **a** conversion rate, **b** hospital stay, **c** bowel movement, **d** pain score, **e** fluid intake

Seven studies reported the conversion rate and showed that SILS had a similar conversion rate as CLS (OR 1.62, 95% CI 0.94 to 2.80, *P* = 0.08; chi-square = 9.01, *P* = 0.17, *I*^2^ = 33%, Fig. 3a). A fixed effects model was used without significant heterogeneity. When BMI > 25, SILS had a higher conversion rate than that of CLS without statistical heterogeneity (OR 2.84, 95% CI 1.38 to 5.87, *P* = 0.005; chi-square = 1.15, *P* = 0. 28, *I*^2^ = 13%, Fig. 3a) (Table 3).

**Table 3:** Comparison of Postoperative data between SILS and CLS for the included studies

Study	Conversion		Bowel movement (day)		Pain score		Hospital stay (day)		Readmission		Fluid intake (day)	
	SILS	CLS	SILS	CLS	SILS	CLS	SILS	CLS	SILS	CLS	SILS	CLS
Curro [9]	0	0	NR	NR	NR	NR	6	6	NR	NR	NR	NR
Yun [10]	1	5	3 ± 1	3 ± 1	NR	NR	8 ± 4	9 ± 5	1	2	NR	NR
Takemasa [11]	1	2	NR	NR	4.3 ± 3.0	5.3 ± 3.5	8 ± 2.3	8.5 ± 2.8	0	0	NR	NR
Suzuki [12]	2	2	NR	NR	NR	NR	7	9	0	0	NR	NR
Yu [13]	NR	NR	2.4 ± 1.0	2.1 ± 1.1	6.4 ± 1.8	6.0 ± 1.8	12.5 ± 2.9	14.6 ± 6.7	NR	NR	3.4 ± 1	3.1 ± 1.2
Tokudka [14]	0	3	4 ± 1.2	4.3 ± 2.1	NR	NR	8.2 ± 7.3	11.2 ± 10.7	NR	NR	NR	NR
Chouillard [15]	32	8	NR	NR	NR	NR	4.9 ± 2.2	5.5 ± 2.3	NR	NR	NR	NR
Kim [16]	1	1	2.7 ± 1.0	3.1 ± 1.2	4.8 ± 1.8	5.9 ± 1.8	6.1 ± 2.4	8.5 ± 5.5	NR	NR	2.7 ± 1.3	3.1 ± 1.2
Song [17]	1	1	NR	NR	4	4	10	10	0	0	NR	NR

*SILS* single-port laparoscopic right hemicolectomy, *CLS* conventional laparoscopic right hemicolectomy, *NR* no record

Compared to the CLS group, the SILS group had a shorter hospital stay (SMD − 0.76, 95%CI − 1.07 to − 0.45, *P* < 0.001; chi-square = 9.85, *P* = 0.08, *I*^2^ = 49%, Fig. 3b) in the fixed effects model.

#### Bowel movement, maximal pain score, and fluid intake

There was no significant difference in bowel movement (SMD − 0.06, 95%CI − 0.38 to 0.26, *P* = 0.72; chi-square = 6.39, *P* = 0.09, *I*^2^ = 53%, Fig. 3c), pain score (SMD − 0.54, 95%CI − 1.59 to 0.51, *P* = 0.32; chi-square = 10.46, *P <* 0.01, *I*^2^ = 81%, Fig. 3d), and fluid intake (SMD − 0.04, 95%CI − 0.72 to 0.65, *P* = 0.92; chi-square = 0.11, *P* = 0.03, *I*^2^ = 79%, Fig. 3e) in the random effects model with statistical heterogeneity.

### Pathological and follow-up outcomes

#### Lymph node dissection, proximal surgical edge (PSE), and distal surgical edge (DSE)

**Fig. 4:**
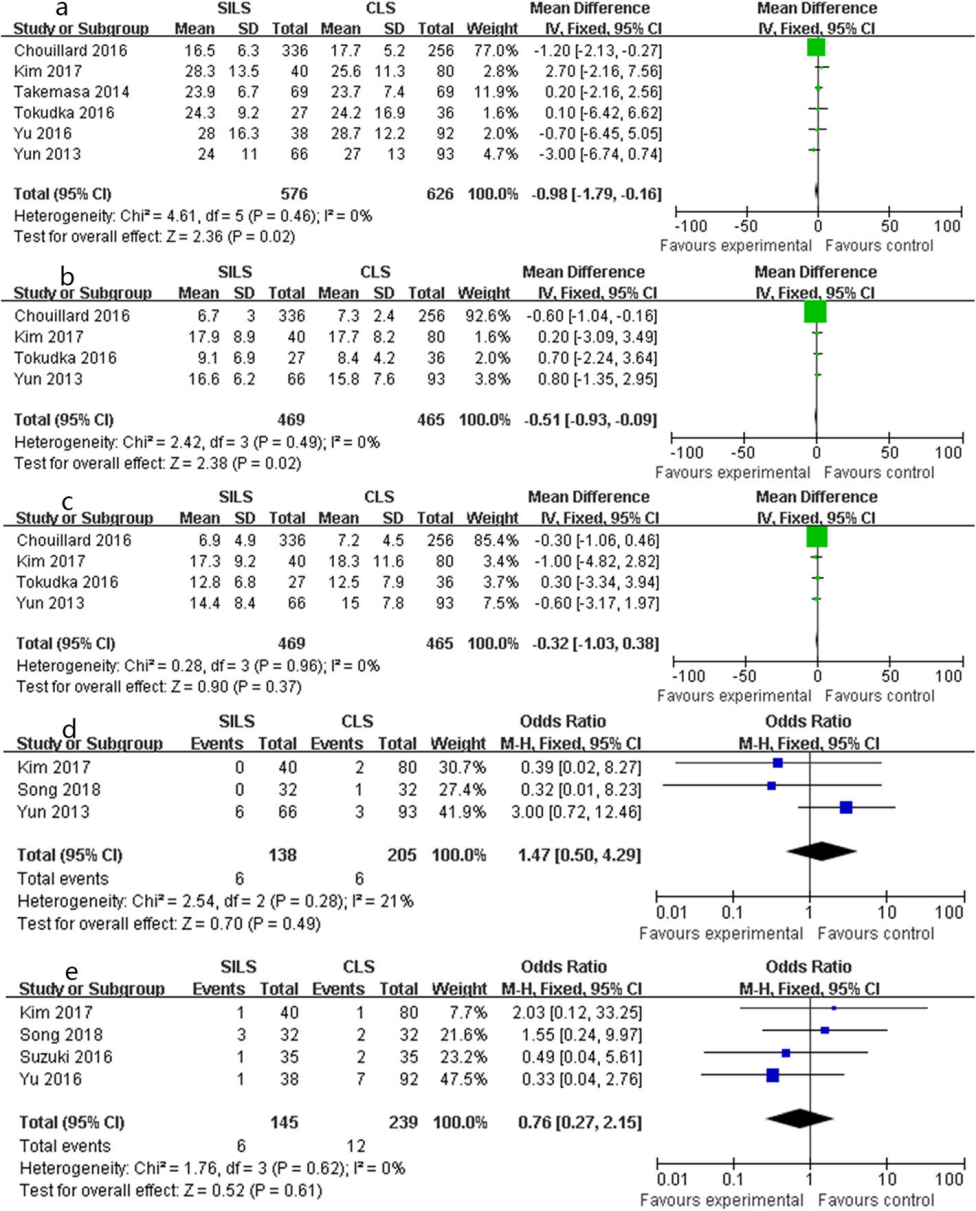
Forest plot of mid-term outcomes: **a** lymph node harvested, **b** proximal surgical edge (DSE), **c** distal surgical edge (DSE), **d** local recurrence, **e** metastasis

SILS had more lymph node dissection (SMD − 0.98, 95%CI − 1.79 to − 0.16, *P* = 0.02; chi-square = 4.61, *P* = 0.46, *I*^2^ = 0%, Fig. 4a) and longer PSE (SMD − 0.51, 95%CI − 0.93 to − 0.09, *P* = 0.02; chi-square = 2.42, *P* = 0.49, *I*^2^ = 0%, Fig. 4b) than CLS in the fixed effects model without huge heterogeneity. The DSE (SMD − 0.32, 95%CI − 1.03 to 0.38, *P* = 0.37; chi-square = 0.28, *P* = 0.96, *I*^2^ = 0%, Fig. 4c) of SILS showed the same result as that of CLS (Table 4).

**Table 4:** Comparison of pathological and follow-up outcomes between SILS and CLS for the included studies

Study	Lymph node resection (*n*)		Proximal surgical edge (cm)		Distal surgical edge (cm)		Recurrence		Metastasis	
	SILS	CLS	SILS	CLS	SILS	CLS	SILS	CLS	SILS	CLS
Curro [9]	25	24	NR	NR	15.5	13	NR	NR	NR	NR
Yun [10]	24 ± 11	27 ± 13	14.4 ± 8.4	15 ± 7.8	16.6 ± 6.2	15.8 ± 7.6	6	3	NR	NR
Takemasa [11]	23.9 ± 6.7	23.7 ± 7.4	NR	NR	NR	NR	NR	NR	NR	NR
Suzuki [12]	16	15	NR	NR	NR	NR	NR	NR	1	2
Yu [13]	28.0 ± 16.3	28.7 ± 12.2	NR	NR	NR	NR	NR	NR	1	7
Tokudka [14]	24.3 ± 9.2	24.2 ± 16.9	12.8 ± 6.8	12.5 ± 7.9	9.1 ± 6.9	8.4 ± 4.2	NR	NR	NR	NR
Chouillard [15]	16.5 ± 6.3	17.7 ± 5.2	6.9 ± 4.9	7.2 ± 4.5	6.7 ± 3.0	7.3 ± 2.4	NR	NR	NR	NR
Kim [16]	28.3 ± 13.5	25.6 ± 11.3	17.3 ± 9.2	18.3 ± 11.6	17.9 ± 8.9	17.7 ± 8.2	0	2	1	1
Song [17]	17	17	8.75	12	10.5	12	0	1	3	2

*SILS* single-port laparoscopic right hemicolectomy, *CLS* conventional laparoscopic right hemicolectomy, *NR* no record

#### Local recurrence and metastasis

SILS had similar local recurrence (OR 1.47, 95% CI 0.50 to 4.29, *P* = 0.49; chi-square = 2.54, *P* = 0.28, *I*^2^ = 21%, Fig. 4d) and metastasis (OR 0.76, 95% CI 0.27 to 2.15, *P* = 0.61; chi-square = 1.76, *P* = 0.62, *I*^2^ = 0%, Fig. 4e) in the fixed effects model without significant heterogeneity.

### Subgroup group analysis

**Fig. 5:**
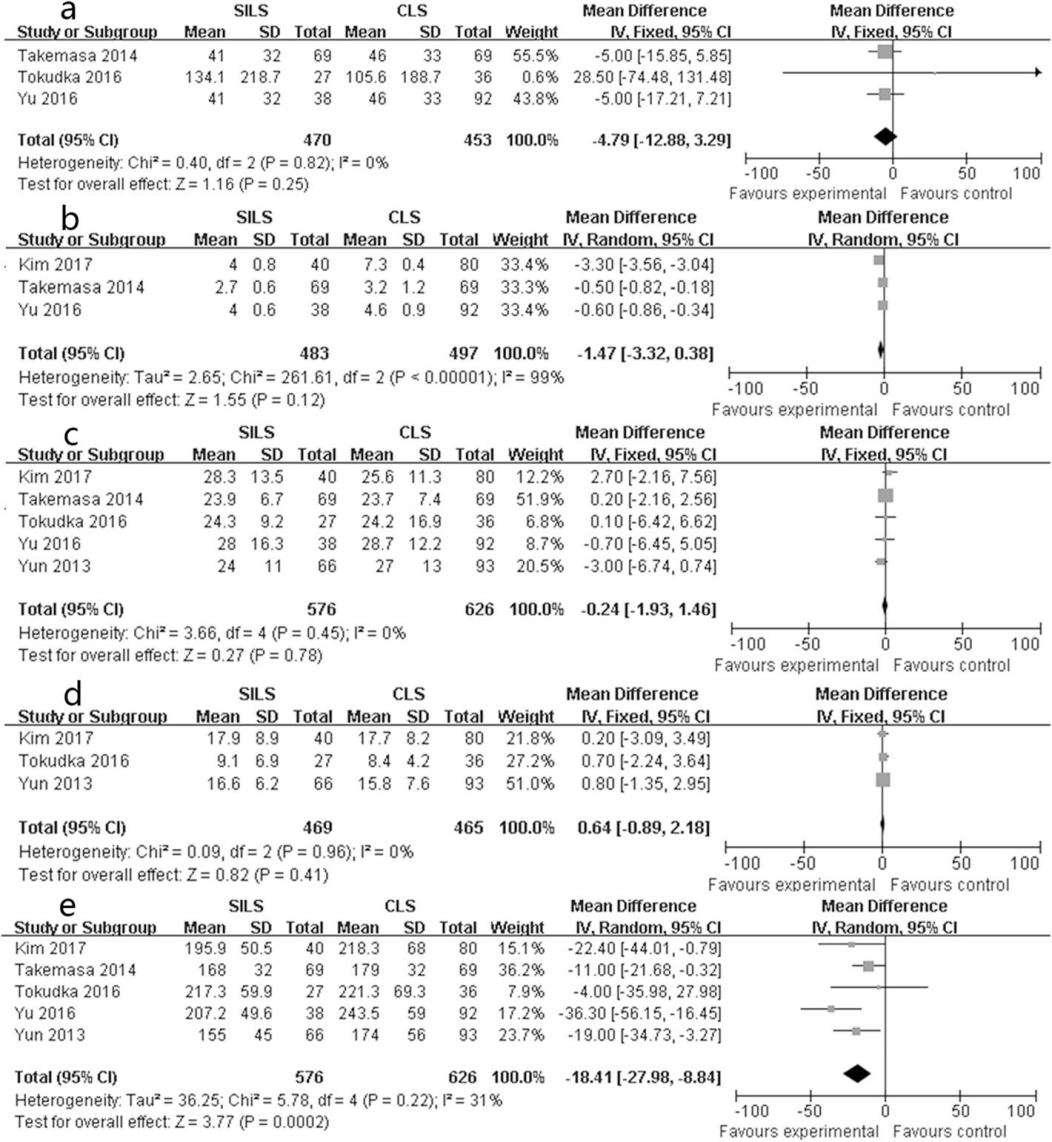
Forest plot of subgroup group: **a** blood loss, **b** incision length, **c** lymph node harvested, **d** distal surgical edge (DSE), **e** operation time

The study by Chouillard contained 592 patients and could have an impact on the results. We removed this study and conducted the meta-analysis again. The results indicated that four indexes were different from the above results. Blood loss (SMD − 4.79, 95% CI − 12.88 to 3.29, *P* = 0.25; chi-square = 1.16, *P* = 0.82, *I*^2^ = 0%, Fig. 5a), incision length (SMD − 1.47, 95% CI − 3.32 to 0.38, *P* = 0.12; chi-square = 261.61, *P* < 0.001, *I*^2^ = 99%, Fig. 5b), lymph node dissection (SMD − 0.24, 95% CI − 1.93 to 1.46, *P* = 0.78; chi-square = 3.66, *P* = 0.45, *I*^2^ = 0%, Fig. 5c), and DSE (SMD 0.64, 95% CI − 0.89 to 2.18, *P* = 0.41; chi-square = 0.09, *P* = 0.96, *I*^2^ = 0%, Fig. 5d) were similar in the two groups.

Other indexes were similar to the previous results, but the operation time (SMD − 18.41, 95% CI − 27.98 to − 8.84, *P* < 0.001; chi-square = 5.78, *P* = 0.22, *I*^2^ = 31%, Fig. 5e) for the SILS group was still better than that for the CLS group.

### Sensitivity analysis

Based on the similar baseline characteristics, Begg’s correlation test (complication, *P* = 0.34) revealed no clear publication bias. The sensitivity analysis showed that the good quality of research would not impact the final results.

## Discussion

In the near future, minimally invasive surgery will become increasingly popular especially in colorectal surgery. This approach could reduce the length of incision and promote the rapid recovery of patients. Medical devices and levels have been developing and improving in recent decades [18]. Currently, an increasing number of laparoscopic surgeries have replaced open surgery. Doctors are also pursuing smaller incisions to solve more problems. Dong et al. had reported that SILS was better than CLS in some data for right colon diseases. We collected the evidence-based data to compare operative, postoperative, pathological, and mid-term outcomes between SILS and CLS in right colon cancer. The good quality of the included studies could provide reliable results.

The results showed that SILS had a shorter operation time and incision length than CLS. The operation time for SILS was 23.49 min shorter than that for CLS and the incision length for SILS was 1.6 cm shorter than that for CLS. Due to the improvement in the device, SILS became easier than before. The cooperation of the surgical term was improved due to the high incidence of malignant tumors, and the surgeon was becoming increasingly skilled. The improved SILS port could contain more instruments, which could reduce the operation duration and incision length. SILS with a shorter operation duration and incision length may accelerate patient recovery, reduce postoperative pain, and promote early activities [19].

SILS had less blood loss than CLS and it could be affected by the surgeons’ experience and surgical skills of the surgeon and other factors. However, intraoperative blood loss was not easy to accurately measure, and the amount of bleeding in the SILS group was only reduced by 8 ml, which would not affect the postoperative recovery of the patient. Therefore, although this indicator was a positive result, it had no clinical significance. SILS also resulted in a shorter hospital stay than that with CLS, and we speculated a short incision could reduce the recovery time of patients and the length of hospital stay. Although the hospital stay after SILS was only shortened by 0.76 days, this effect shows a trend, and we hope that the hospital stay may be shortened significantly in the future. SILS had the advantages of incision length, blood loss, operation duration, and hospital stay. Bowel movement and pain score appeared to be the same in the two groups herein, but these indexes were not the same in the relevant studies [20].

Due to obesity, vascular variation, narrow pelvis, and heavy mesentery, SILS would be more difficult than CLS [21]. The main reason for the high conversion rate was abdominal adhesion, especially in patients with a previous abdominal history (23.3% patients had an abdominal history). We found that the conversion rate of the SILS group with large BMI was higher than that of the CLS group, especially for patients with a large BMI (BMI > 25).

The complication rate was similar in the two groups. Three studies reported complication (C-D grade), and there was no difference between the two groups. We suspected that this effect was related to the suitable cases for SILS chosen by the doctor, and appropriate treatment options facilitated the operation smoothly and reduced the incidence of complications. Through the development of medical devices and technological advancements, the skills of the surgeon have become increasingly sophisticated with the increasing number of surgical procedures. Thus, the complication rate of SILS was similar to that of CLS [22]. The complication rate was the main contributor to surgical technique and operation time.

Due to the large specimen of the right colon, CLS needed to extend the umbilical incision to facilitate specimen removal. However, SILS with the single long incision in the umbilicus might be more convenient to remove specimens without increasing the length. SILS with a short incision length might reduce postoperative pain, promote early activities, and reduce the incidence of complication.

Six studies reported the type of ileocolonic anastomosis, and these studies were all extra-corporeal anastomoses. Three studies were end-to-end ileocolonic anastomoses, and three studies were end-to-side ileocolonic anastomoses. Lymph node dissection and PSE of SILS were better than those of CLS, and DSE tended to be the same in the two groups. In SILS, which had a larger incision than CLS, removing more of the intestine from the abdominal wall was easier and the surgical margin was better than those in CLS. Due to the short development time of SILS, a lack of clinical data might impact the results of distal metastasis and produce bias. We expected more clinical studies to further illuminate the relationship between the groups.

We removed a large study representing almost half of all patients and performed a meta-analysis again. The results indicated that four indexes were different from the above results. Blood loss, incision length, lymph node dissection, and DSE were similar in the two groups. After repeating the statistical analysis, the operation time in the SILS group was still better than that in the CLS group. Due to the large size of the single study, it may cause potential selection bias and affect the results of the study. After repeating the statistical analysis, the results of lymph node dissection, PSE, and DSE were similar to previous research [23].

In addition, the results of this study might have several limitations. First, nine studies only contained a modest number of patients and all the included studies were not of the highest quality of evidence. All the included studies were comparative nonrandomized clinical trials, and no RCTs were included due to short development times. Second, one study contained almost half of all patients and affected the results. Third, different patient conditions and medical facilities could cause potential selection bias. In this study, there were only two European studies and fewer people with a large BMI. This potential selection bias could affect the final results. Finally, the long-term follow-up results were incomplete due to the short development of SILS. Only a few articles had reported recurrence and metastasis data between the two techniques, which would affect the follow-up outcomes. Furthermore, RCTs with long follow-up outcomes were necessary to compare SILS with CLS for patients.

## Conclusions

In summary, this study had compared the reliability and safety of the SILS and CLS for the treatment of right colon cancer. SILS had a shorter operation time, shorter hospital stay, shorter incision length, less blood loss, better lymph node dissection, and PSE than CLS. Complication, conversion, follow-up outcomes, and other data were similar between SILS and CLS. After we removed the large study, we performed the meta-analysis again. The operation time in the SILS group was still shorter than that in the CLS group. With the continuous development of professional technology, future evidence of improvements in the long-term outcomes might justify the advantages and disadvantages of SILS and CLS for treating right colon cancer. SILS had the advantages of the only long incisions to remove specimens conveniently without increasing the incision length. Therefore, we propose that SILS could be a feasible model for right colon cancer.

## Supplementary information

**Additional file 1:** **Table S1.** The detailed retrieval strategy in PubMed database.

## Data Availability

All data generated or analyzed during this study are included in this published article.

## Abbreviations

BMI: Body mass index
CLS: Conventional laparoscopic right hemicolectomy
DSE: Distal surgical edge
NRCT: Non-randomized comparative trial
OR: Odds ratios
OS: Overall survival
PSE: Proximal surgical edge
SILS: Single-incision laparoscopic right hemicolectomy
SMD: Standardized mean difference
