# Supplementary material for: Systematic review of comparing single-incision versus conventional laparoscopic right hemicolectomy for right colon cancer
Source: World J Surg Oncol. 2019 Nov 4;17:179. doi: 10.1186/s12957-019-1721-6 (PMC6829956; doi:10.1186/s12957-019-1721-6)
Supplement: Supplementary file 1 — Additional file 1: Table S1. The detailed retrieval strategy in PubMed database. [file 12957_2019_1721_MOESM1_ESM.doc]

**The detailed retrieval strategy in PubMed database.**

Search details:((((single-incision[All Fields] OR single-site[All Fields]) OR single-port[All Fields]) AND ("colonic neoplasms"[MeSH Terms] OR ("colonic"[All Fields] AND "neoplasms"[All Fields]) OR "colonic neoplasms"[All Fields] OR ("colon"[All Fields] AND "cancer"[All Fields]) OR "colon cancer"[All Fields])) OR (right[All Fields] AND ("colonic neoplasms"[MeSH Terms] OR ("colonic"[All Fields] AND "neoplasms"[All Fields]) OR "colonic neoplasms"[All Fields] OR ("colon"[All Fields] AND "cancer"[All Fields]) OR "colon cancer"[All Fields]))) AND ("laparoscopy"[MeSH Terms] OR "laparoscopy"[All Fields] OR ("laparoscopic"[All Fields] AND "surgery"[All Fields]) OR "laparoscopic surgery"[All Fields])
